# Chronic Ultraviolet Irradiation to the Skin Dysregulates Adrenal Medulla and Dopamine Metabolism In Vivo

**DOI:** 10.3390/antiox10060920

**Published:** 2021-06-07

**Authors:** Hye-Sun Lim, Kyeong-No Yoon, Jin Ho Chung, Yong-Seok Lee, Dong Hun Lee, Gunhyuk Park

**Affiliations:** 1 Herbal Medicine Resources Research Center, Korea Institute of Oriental Medicine, 111 Geonjae-ro, Naju 58245, Korea; qp1015@kiom.re.kr; 2 Department of Biomedical Sciences, Graduate School, Seoul National University, Seoul 03080, Korea; kyeongnoyoon@naver.com (K.-N.Y.); jhchung@snu.ac.kr (J.H.C.); 3 Department of Dermatology, Seoul National University College of Medicine, Seoul 03080, Korea; 4 Medical Research Center, Institute of Human-Environment Interface Biology, Seoul National University, Seoul 03080, Korea; 5 Institute on Aging, Seoul National University, Seoul 03080, Korea; 6 Department of Physiology, Neuroscience Research Institute, Seoul National University College of Medicine, Seoul 03080, Korea; yongseok7@snu.ac.kr; 7 Department of Biomedical Sciences, Neuroscience Research Institute, Seoul National University College of Medicine, Seoul 03080, Korea

**Keywords:** UVB, adrenal glands, chromaffin cell, dopamine, DβH, oxidative damage

## Abstract

Ultraviolet (UV) radiation has a strong biological effect on skin biology, and it switches on adaptive mechanisms to maintain homeostasis in organs such as the skin, adrenal glands, and brain. In this study, we examined the adaptation of the body to repeated bouts of UVB radiation, especially with respect to the catecholamine synthesis pathway of the adrenal glands. The effects of UVB on catecholamine-related enzymes were determined by neurochemical and histological analyses. To evaluate catecholamine changes after chronic excessive UVB irradiation of mouse skin, we examined dopamine and norepinephrine levels in the adrenal glands and blood from UV-irradiated and sham-irradiated mice. We found that chronic excessive UVB exposure significantly reduced dopamine levels in both tissues but did not affect norepinephrine levels. In addition, UVB irradiation significantly increased the levels of related enzymes tyrosine hydroxylase and dopamine-β-hydroxylase. Furthermore, we also found that apoptosis-associated markers were increased and that oxidative defense proteins were decreased, which might have contributed to the marked structural abnormalities in the adrenal medullas of the chronically UVB-irradiated mice. This is the first evidence of the damage to the adrenal gland and subsequent dysregulation of catecholamine metabolism induced by chronic exposure to UVB.

## 1. Introduction

Humans are constantly confronted with various stressors, and physical responses and adaptation to these stressors are essential for health [1]. The adrenal gland is important for responding to physiological challenges and can adapt its activity to varying physiological needs [1,2]. Appropriative adaptation is essential because dysregulation of responses to stress causes various disorders [1]. The adrenal gland, which is a critical controller of these responses and body homeostasis, is divided into the cortex and medulla regions [1]. The adrenal cortex produces mineralocorticoids, glucocorticoids, and androgens [1]. The steroids aldosterone and cortisol produced in the adrenal cortex help regulate mineral balance, glucose metabolism, immune system suppression, masculinization, and homeostasis maintenance in response to stress [1,2]. The inner medulla produces catecholamines, which induce a rapid response throughout the body in stressful situations.

Ultraviolet (UV) radiation has powerful effects on skin biology and on diverse vitamin D-dependent and vitamin D-independent regulatory pathways involved in immune homeostasis [3]. Moreover, the skin adapts to UVB exposure through cutaneous and central hypothalamic–pituitary–adrenal (HPA) axis activity to maintain homeostasis in the adrenal glands, skin, and brain [4,5,6]. Moderate UVB exposure enhances learning and memory by inducing glutamate synthesis, synaptic vesicle packaging, and glutamate release in the cortical and hippocampal regions of the brain [7]. Although humans can fully adapt to moderate UVB radiation and even benefit from it, excessive UVB radiation has negative effects. In general, excessive UVB irradiation causes stress and potentially leads to skin aging, sunburn, and skin carcinogenesis [8]. Skin exposure to chronic excessive UVB inhibits neurogenesis and synaptic plasticity by over-activating the glucocorticoid signaling pathway [8]. Furthermore, chronic excessive repeated UVB irradiation results in depressive behavior due to hippocampal dysfunction caused by HPA axis hyperactivity [8]. Although UVB has adverse effects on distant organs, the mechanisms utilized in the adrenal glands to adapt to UVB-induced stress have not been reported. For the first time, we show here the adverse effects of UVB irradiation on catecholamine responses and enzymatic reactions in the blood and adrenal glands. Moreover, we demonstrate that UVB irradiation causes apoptotic damage and suppresses oxidative defense response signaling in the adrenal glands.

## 2. Materials and Methods

### 2.1. Animals

Female hairless mice (CrlOri:SKH1, 5 weeks old) were purchased from the Orient Experimental Animal Breeding Center (Seoul, Korea). The animals were maintained under temperature- and light-controlled conditions (20–23 °C, 12-h light/dark cycle) with food and water provided ad libitum. The experimental protocol and design of the study were approved by the Committee on Animal Care of the Korea Institute of Oriental Medicine (KIOM; Approval No. KIOM-20-071 and KIOM-21-021).

### 2.2. UVB Irradiation

Mice were assigned to one of nine groups that represented three separate experimental sets: (A1) control (n = 10); (A2) UVB (n = 10); (B1) control (n = 8); (B2) UVB (n = 8); (B3) UVB + disulfiram (n = 8); (B4) UVB + nepicastat (n = 8); (C1) control (n = 5); (C2) UVB (n = 5); (C3) UVB + Nrf2 activator (CDDO-Me; R&D systems; #6646, n = 4); (C4) UVB + Nrf2 activator + nepicastat (n = 5). Irradiation was performed using TL20W/12RS UVB lamps (Philips, Eindhoven, the Netherlands) with an emission spectrum between 275 and 320 nm. UVC wavelengths (<290 nm) were blocked by a UVC cut filter (T_max_: 290–315 nm; cutoff: 280 nm or less) placed 3.5 cm in front of the UV lamp. Intensity of the UV radiation was measured using a UV radiometer (97-0015-02, UVP Radiometer, Analytik Jena, Upland, CA, USA). Briefly, we subjected the anesthetized mice to chronic excessive UVB irradiation by exposing their dorsal skin to UV light at 100–300 mJ/cm^2^ for 3 days per week (Monday, Wednesday, and Friday) for 10 weeks. This method is a commonly used skin aging model that triggers aging-like changes in the epidermal thickness (Supplementary Figure S1). The treatment protocol is described in Supplementary Table S1. Then, either disulfiram or nepicastat, dissolved in normal saline, was administered for 3 days per week (Monday, Wednesday, and Friday) for 10 weeks to selected treatment groups.

### 2.3. Immunohistochemistry, Immunoblotting, and Enzyme-Linked Immunosorbent Assay (ELISA)

For immunoblotting, HPLC, and ELISA, the adrenal glands were rapidly dissected and homogenized using standard laboratory techniques [8,9,10,11,12,13]. The final supernatant was stored at −80 °C until further use. Then, we performed immunoblotting for dopamine β-hydroxylase (DβH; 1:1000 dilution; ImmunoStar, Hudson, WI, USA), and the apoptotic markers cleaved caspase-7 (1:500 dilution), caspase-9 (1:100 dilution), cleaved caspase-9 (1:1000 dilution), caspase-3 (1:500 dilution), and cleaved caspase-3 (1:2000 dilutions; all from Cell Signaling Technology, Danvers, MA, USA) as previously described [8,9]. The levels of the catecholamines dopamine and norepinephrine (both from Rocky Mountain Diagnostics, Colorado Springs, CO, USA), and that of DβH (Aviva Systems Biology, San Diego, CA, USA), were quantified in the adrenal glands or serum of the mice using commercially available ELISA kits according to the manufacturers’ protocols [10,13]. In addition, the catecholamines were subjected to HPLC analysis as previously described [9,10]. Moreover, the levels of cleaved caspase-7 (Ray Biotech, Norcross, GA, USA), -9 (Novus biologicals, Littleton, CO, USA), and -3 (Cell Signaling Technology, Danvers, MA, USA) were quantified in the adrenal glands of the mice using commercially available ELISA kits according to the manufacturers’ protocols. 

### 2.4. Statistical Analyses

All statistical analyses were performed using GraphPad Prism 7.0 (GraphPad Software, Inc., San Diego, CA, USA). Values are expressed as the mean ± standard error of the mean (SEM). Statistical comparisons among different treatments were performed using the *t*-test followed by the Mann–Whitney test. Values were considered to be significantly different if *p* < 0.05.

## 3. Results and Discussion

**Figure 1:**
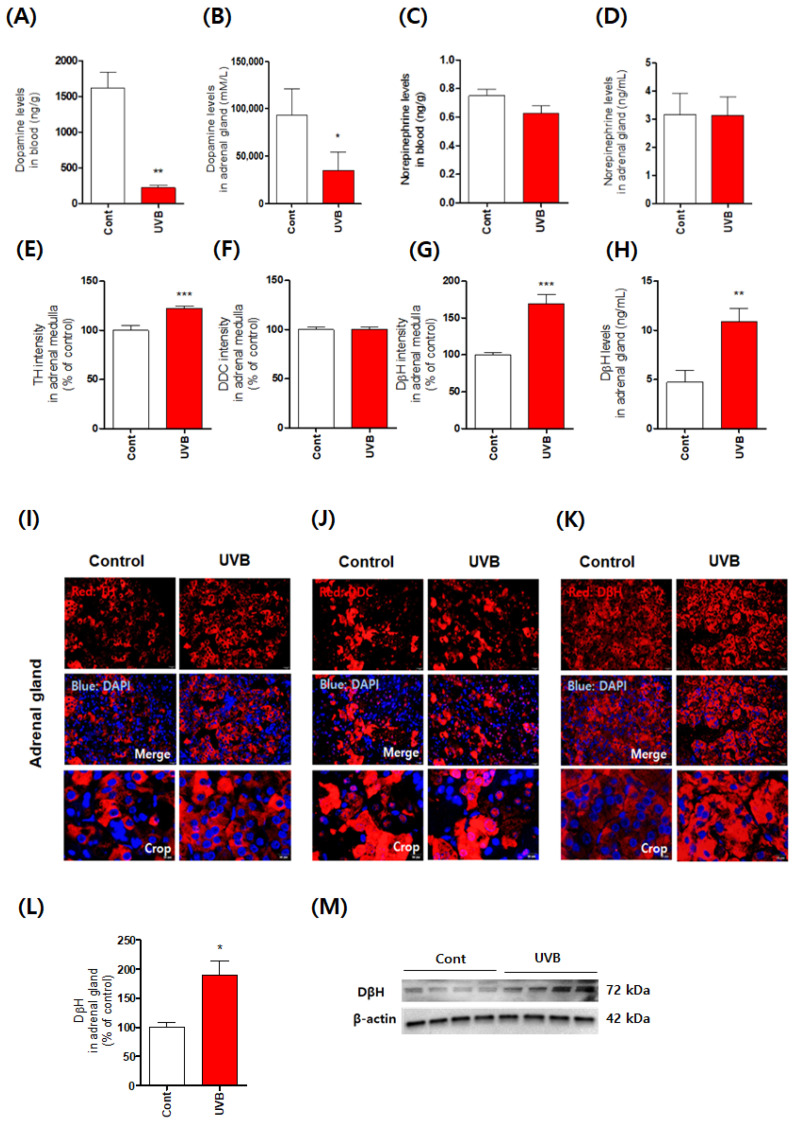
Acceleration of stress response via the upregulation of dopamine-β-hydroxylase (DβH) expres-sion in the adrenal medulla caused by mouse skin exposure to chronic excessive UVB irradia-tion. Dopamine (**A**,**B**) and norepinephrine (**C**,**D**) levels in the blood and adrenal gland were quantified using ELISA kits and HPLC-ECD. The levels of the related enzymes, tyrosine hydrox-ylase (TH; (**E**)), L-DOPA decarboxylase (DDC; (**F**)), and dopamine β-hydroxylase (DβH; (**G**)), were quantified by determining fluorescence intensity using immunohistochemistry. The levels of the DβH (**H**) were determined by ELISA. Representative photomicrographs are shown (**I**–**K**). DβH levels were also quantified using ELISA (**L**) and western blotting (**M**). Values represent the mean ± standard error of the mean. Statistical significance of differences is illustrated as follows: * *p* < 0.05, ** *p* < 0.01, and *** *p* < 0.001 compared with the control group.

Adaptation responses to stress are achieved by two main mechanisms [14]. The sympathetic nervous system controls the stress response through the hypothalamus. Stressful stimulation causes the hypothalamus to send signals to the adrenal medulla (sympatho-adrenomedullary; SA system) and adrenal cortex via nerve impulses, mediating stress responses through adrenocorticotropic hormone (ACTH) produced by the anterior pituitary. When presented with a stressful situation, the body responds by releasing epinephrine and norepinephrine in the adrenal medulla. However, during long-term stress responses (HPA system), the hypothalamus triggers ACTH release from the anterior pituitary. ACTH stimulates the adrenal cortex to secrete corticosterone. This reaction facilitates homeostasis, i.e., adaptation responses to the stress stimulus, and recovery [15]. The HPA axis stress response to UVB stimuli has been previously described. According to Slominski et al., exposure of the skin to UVB upregulated the levels of urocortins, β-endorphin, and corticosterone in the skin and blood. In addition, this treatment raised the levels of corticotrophin releasing hormone (CRH) and ACTH in blood as well as mRNA and protein expression levels of CRH in the hypothalamus and of induced melanocortin receptor type 2, steroidogenic acute regulatory protein, and steroid 11b-hydroxylase in the adrenal glands [4,5]. Moreover, hypophysectomy eliminated stimulatory effects of UVB on blood levels of ACTH and corticosterone, but not on the levels of cutaneous corticosteroids. Further, hypophysectomy did not affect upregulation of CRH and urocotin levels by UVB, so the regulation of homeostasis by UVB via the HPA axis requires a healthy pituitary gland for systemic effect [5,6]. The effect of UV stimulation depends on the wavelength with the magnitude of effect decreasing in the following order: UVC > UVB > UVA. For example, UVA irradiation causes only weak physiological and biochemical alterations, limited mostly to increases in CRH and β-endorphin levels [6]. The HPA axis has been shown to play an important role in the response of the skin and adrenal glands to UVB irradiation and maintenance of body homeostasis. However, few studies have shown a link between UVB effects and the SA system. In particular, no studies have reported on the changes in the SA system after long-term UVB stimulation. Thus, in this study, we focused on SA system responses to the stress induced by UVB. We first investigated the levels of the catecholamines norepinephrine and dopamine in the blood and adrenal glands (Figure 1). Norepinephrine is synthesized from the amino acid tyrosine through a series of enzymatic steps (tyrosine → levodopa (L-DOPA) → dopamine → norepinephrine) in the SA system [16]. The conversion of tyrosine to L-DOPA is carried out by tyrosine hydroxylase (TH) [16]. L-DOPA is converted to dopamine by DOPA decarboxylase. Dopamine is then converted to norepinephrine by DβH [16,17]. Whereas the conversion (tyrosine → dopamine) occurs predominantly in the cytoplasm, the conversion of dopamine to norepinephrine mediated by DβH occurs mainly inside neurotransmitter vesicles [16]. Surprisingly, in this study, dopamine levels were significantly reduced by UVB irradiation, but norepinephrine levels were not altered. Moreover, TH and DβH levels increased significantly compared to those of other enzymes. In addition, the levels of monoamine oxidase and catechol-*O*-methyltransferase, which are related to dopamine metabolism, were not significantly changed by UVB treatment (Supplementary Figure S2). Consequently, the adrenal gland, damaged by UVB, was likely activated as part of a stress response to increase norepinephrine using a mechanism that increases DβH expression. We speculated that this phenomenon included a change in the catecholamine production mechanism induced by stress response compensation to repeated adaptation reactions mediated by chronic excessive repeated UVB irradiation.

**Figure 2:**
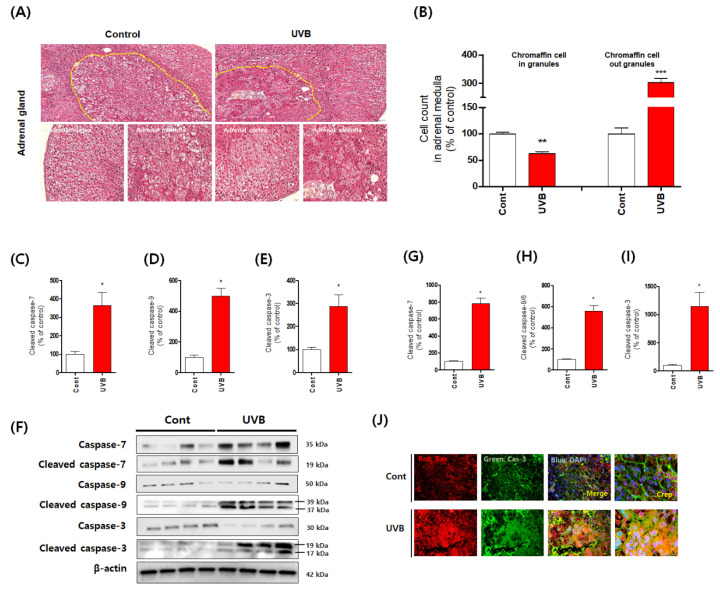
Accelerated cytotoxicity and augmented apoptosis signaling in chromaffin cells of the adrenal medulla induced by mouse skin exposure to chronic excessive UVB irradiation. (**A**) Representative histological analysis of an adrenal gland section damaged by the exposure to UVB. (**B**) Hematoxylin/eosin staining was used to identify chromaffin cell counts in granules. (**C**–**E**) Western blotting analysis of the apoptosis markers cleaved caspase-7 (**C**), cleaved caspase-9 (**D**), and cleaved caspase-3 (**E**). (**F**) Representative western blot images are shown. (**G**–**I**) The levels of cleaved caspase-7 (**G**), cleaved caspase-9/6 (**H**), and cleaved caspase-3 (**I**) were determined by ELISA. (**J**) Expression levels of Bax and cleaved caspase-3 were confirmed by using immunohistochemistry. Values represent the mean ± standard error of the mean. Statistical significance of differences is illustrated as follows: * *p* < 0.05, ** *p* < 0.01, and *** *p* < 0.001 compared with the control group.

**Figure 3:**
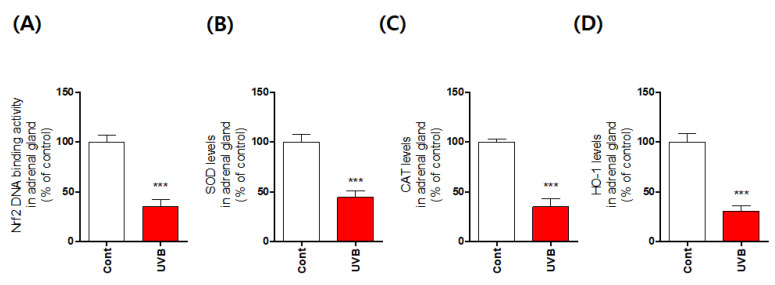
Reduction in expression levels of oxidative defense enzymes in the adrenal medulla induced by mouse skin exposure to chronic excessive UVB. The levels of Nrf2 (**A**), SOD (**B**), CAT (**C**), and HO-1 (**D**) were quantified using ELISA kits. Values represent the mean ± standard error of the mean. *** *p* < 0.001 compared with the control group.

**Figure 4:**
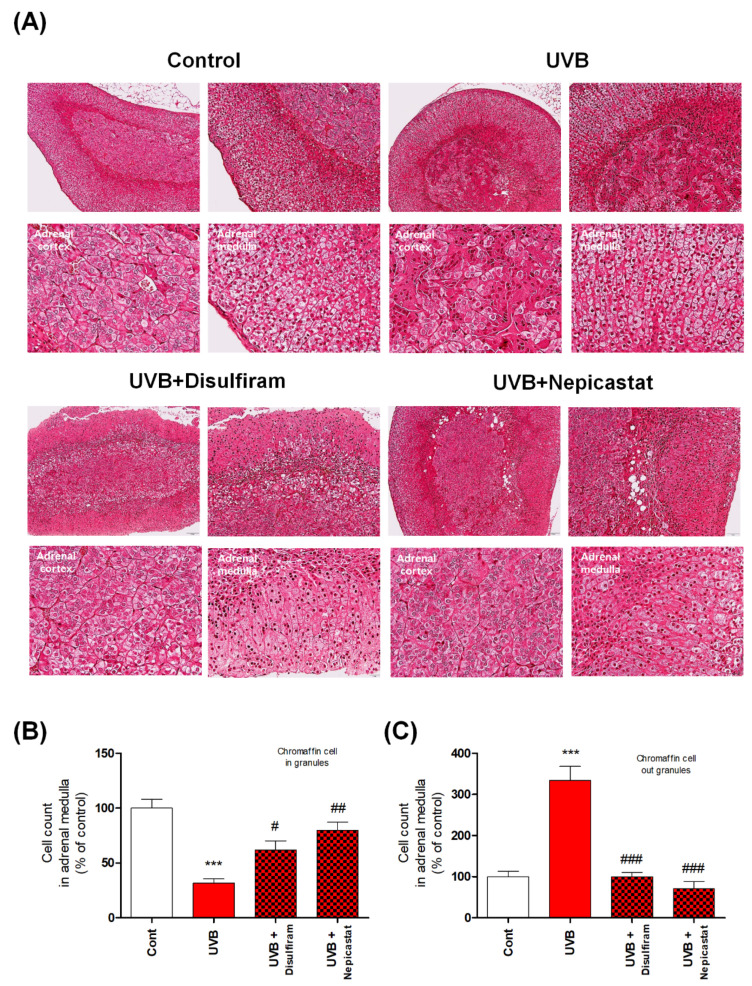
Protective effects of dopamine-β-hydroxylase (DβH) inhibitors against cytotoxic damage to the chromaffin cells of the adrenal medulla caused by skin exposure to chronic excessive UVB. (**A**) Representative histological analysis of an adrenal gland section damaged by exposure to UVB. (**B**,**C**). H&E staining was used to identify chromaffin cell counts in granules. Values represent the mean ± standard error of the mean. Statistical significance of differences is illustrated as follows: *** *p* < 0.001 compared with the control group and # *p* < 0.05, ## *p* < 0.01, and ### *p* < 0.001 compared with the UVB group.

**Figure 5:**
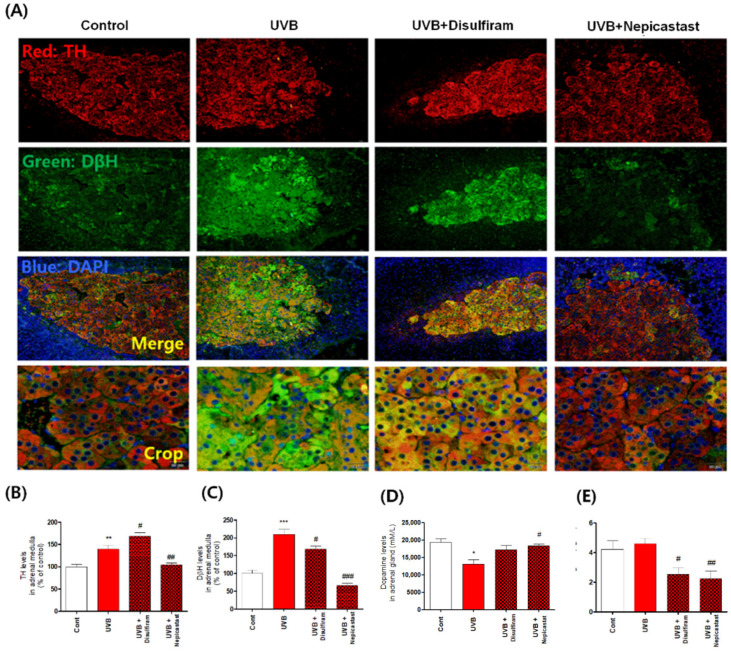
Effects of dopamine-β-hydroxylase (DβH) inhibitors on dopamine and norepinephrine levels in the adrenal medulla following skin exposure to chronic excessive UVB irradiation. (**A**–**C**) The levels of tyrosine hydroxylase (TH; (**A**,**B**) and DβH (**A**,**C**) were quantified by determining fluorescence intensity using immunohistochemistry. Dopamine (**D**) and norepinephrine (**E**) levels were quantified using ELISA kits. Values represent the mean ± standard error of the mean. Statistical significance of differences is illustrated as follows: * *p* < 0.05, ** *p* < 0.01, and *** *p* < 0.001 compared with the control group and # *p* < 0.05, ## *p* < 0.01, and ### *p* < 0.001 compared with the UVB group.

**Figure 6:**
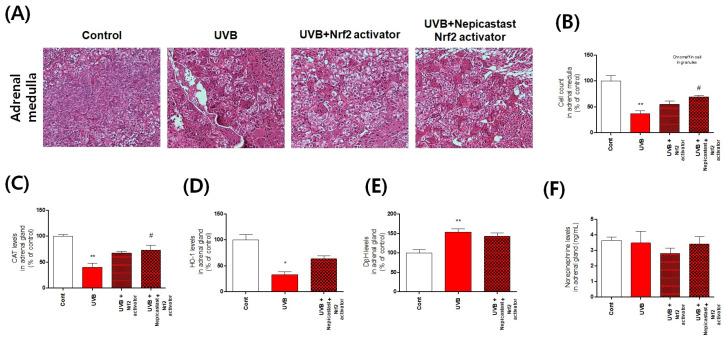
Effects of the dopamine-β-hydroxylase (DβH) inhibitor nepicastat and Nrf2 activator on various indices in the adrenal medulla following mouse skin exposure to chronic excessive UVB irradiation. (**A**) Representative histological analysis of an adrenal gland section damaged by exposure to UVB. (**B**) Effects of nepicastat and of its combination with the Nrf2 activator on chromaffin cell counts in granules revealed by hematoxylin/eosin staining. (**C**–**F**) ELISA-based quantification of the levels of the Nrf2-related enzymes CAT (**C**) and HO-1 (**D**), and of DβH (**E**) and norepinephrine (**F**). Values represent the mean ± standard error of the mean. Statistical significance of differences is illustrated as follows: * *p* < 0.05 and ** *p* < 0.01 compared with the control group and # *p* < 0.05 compared with the UVB group.

The catecholamines dopamine and norepinephrine are primarily produced by the chromaffin cells in the adrenal medulla [1]. Cluster-arranged chromaffin cells store catecholamines in secretory vesicles (chromaffin granules) [1,18]. Sustentacular cells are located at the peripheries of these clusters, whereas ganglion cells are found individually or in interfering clusters among the chromaffin cells or nerve fibers [1,18,19]. The rapid stress reaction causes the release of the catecholamines epinephrine and norepinephrine from the chromaffin cells [19]. Therefore, it is very important to identify the phenotype of the chromaffin cells of the adrenal medulla. Thus, we confirmed this phenotype using histological analysis. We observed that chromaffin cells of UVB-irradiated mice were damaged by UVB irradiation (Figure 2). In particular, the shape of chromaffin granules, which are responsible for the synthesis and storage of catecholamine, was changed and their size was significantly reduced. We also found evidence of enhanced apoptosis, reflected specifically in statistically significant increases in the levels of cleaved caspases 3, 7, and 9 (Figure 2). To further confirm that the apoptosis signal was accompanied by oxidative stress, we measured expression levels of oxidative stress-response molecules, such as nuclear factor erythroid-2-related factor 2 (Nrf2), superoxide dismutase, catalase, and heme oxygenase 1, and found that their expression was significantly reduced (Figure 3). Furthermore, we anticipated that the rapid increase in DβH levels would have an adverse impact and hypothesized that the chronic excessive increase in DβH was a possible factor in the toxicity of UVB irradiation to the chromaffin cells. We verified this assumption by investigating the effects of the DβH inhibitors disulfiram and nepicastat. Indeed, DβH inhibitors reduced cytotoxic damage to the chromaffin cells in the adrenal glands (Figure 4 and Figure 5). However, although the toxic damage to the adrenal glands decreased, further ways of targeting this organ are necessary, as stress response control for the reduction of norepinephrine is expected to decrease. As a preliminary experiment, we found that the combined administration of a DβH inhibitor and an Nrf2 activator not only reduced toxic damage to the adrenal glands but also restored norepinephrine levels (Figure 6). However, further research to explain this phenomenon will be necessary in the future. Here, we report new experimental evidence of this phenomenon and believe that more research should be conducted, as this mechanism is different from the stress responses reported to date.

## 4. Conclusions

**Figure 7:**
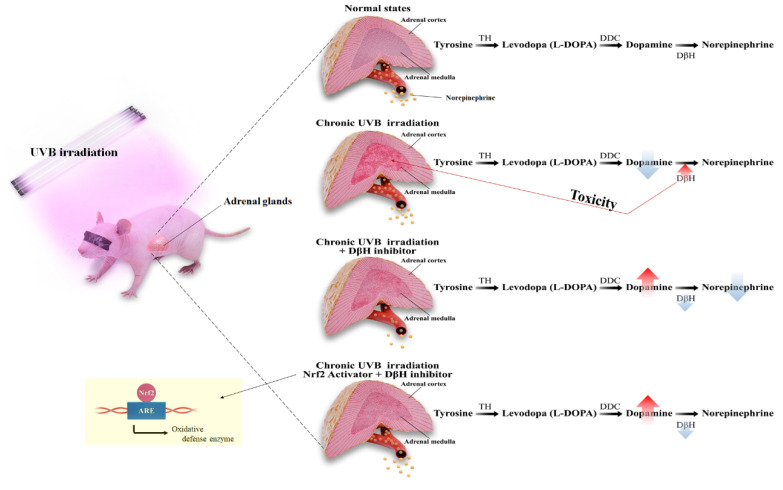
Schematic of the mechanism proposed for chronic ultraviolet irradiation to the skin dysregulating adrenal medulla and dopamine metabolism.

In summary, chronic excessive repeated UVB stimulation-induced norepinephrine was associated with characteristic increases in the expression of DβH, which is involved in dopamine metabolism, and is considered an equilibrium condition caused by adrenal gland damage (Figure 7).

## Data Availability

The data presented in this study are available on request from the corresponding author.

## Supplementary Materials

The following are available online at https://www.mdpi.com/article/10.3390/antiox10060920/s1, Figure S1: Accelerated skin aging in mice induced by skin exposure to chronic excessive UVB irradiation, Figure S2: Lack of changes in the levels of dopamine metabolism-related enzymes following mouse skin exposure to chronic excessive UVB irradiation, Table S1: UVB irradiation schedule.

## References

[1] Berger I., Werdermann M., Bornstein S.R., Steenblock C. (2019). The adrenal gland in stress—Adaptation on a cellular level. J. Steroid Biochem. Mol. Biol..

[2] Sunwoo S.H., Lee J.S., Bae S., Shin Y.J., Kim C.S., Joo S.Y., Choi H.S., Suh M., Kim S.W., Choi Y.J. (2019). Chronic and acute stress monitoring by electrophysiological signals from adrenal gland. Proc. Natl. Acad. Sci. USA.

[3] Abhimanyu A., Coussens A.K. (2017). The role of UV radiation and vitamin D in the seasonality and outcomes of infectious disease. Photochem. Photobiol. Sci..

[4] Slominski A.T. (2015). Ultraviolet radiation (UVR) activates central neuro-endocrine-immune system. Photodermatol. Photoimmunol. Photomed..

[5] Skobowiat C., Slominski A.T. (2015). UVB activates hypothalamic–pituitary–adrenal axis in C57BL/6 mice. J. Investig. Dermatol..

[6] Slominski A.T., Amijewski M.A., Plonka P.M., Szaflarski J.P., Paus R. (2018). How UV Light Touches the Brain and Endocrine System Through Skin, and Why. Endocrinology.

[7] Zhu H., Wang N., Yao L., Chen Q., Zhang R., Qian J., Hou Y., Guo W., Fan S., Liu S. (2018). Moderate UV exposure enhances learning and memory by promoting a novel glutamate biosynthetic pathway in the brain. Cell.

[8] Han M., Ban J.-J., Bae J.-S., Shin C.-Y., Lee N.H., Chung J.H. (2017). UV irradiation to mouse skin decreases hippocampal neurogenesis and synaptic protein expression via HPA axis activation. Sci. Rep..

[9] Park G., Lee S.H., Oh D.-S., Kim Y.-U. (2017). Melatonin inhibits neuronal dysfunction-associated with neuroinflammation by atopic psychological stress in NC/Nga atopic-like mouse models. J. Pineal Res..

[10] Lim H.-S., Moon B.C., Lee J., Choi G., Park G. (2020). The insect molting hormone 20-hydroxyecdysone protects dopaminergic neurons against MPTP-induced neurotoxicity in a mouse model of Parkinson’s disease. Free Radic. Biol. Med..

[11] Park G., Moon B.C., Oh D.S., Kim Y.U., Park M.K. (2021). Enhanced Nrf2 up-regulation by extracellular basic pH in a human skin equivalent system. J. Cell. Mol. Med..

[12] Lim H.-S., Kim J.-S., Moon B.C., Ryu S.M., Lee J., Park G. (2019). Batryticatus Bombyx Protects Dopaminergic Neurons against MPTP-Induced Neurotoxicity by Inhibiting Oxidative Damage. Antioxidants.

[13] Lim H.S., Kim J.S., Moon B.C., Choi G., Ryu S.M., Lee J., Ang M.J., Jeon M., Moon C., Park G. (2019). Cicadidae Periostracum, the Cast-Off Skin of Cicada, Protects Dopaminergic Neurons in a Model of Parkinson’s Disease. Oxidative Med. Cell. Longev..

[14] Galluzzi L., Yamazaki T., Kroemer G. (2018). Linking cellular stress responses to systemic homeostasis. Nat. Rev. Mol. Cell Biol..

[15] Van Bodegom M., Homberg J.R., Henckens M.J. (2017). Modulation of the hypothalamic-pituitary-adrenal axis by early life stress exposure. Front. Cell. Neurosci..

[16] Miyajima K., Kawamoto C., Hara S., Mori-Kojima M., Ohye T., Sumi-Ichinose C., Saito N., Sasaoka T., Metzger D., Ichinose H. (2021). Tyrosine hydroxylase conditional knockout mice reveal peripheral tissue-dependent differences in dopamine biosynthetic pathways. J. Biol. Chem..

[17] Catelas D.N., Serrão M.P., Soares-Da-Silva P. (2020). Effects of nepicastat upon dopamine-β-hydroxylase activity and do-pamine and norepinephrine levels in the rat left ventricle, kidney, and adrenal gland. Clin. Exp. Hypertens..

[18] Vollmer R.R., Baruchin A., Kolibal-Pegher S.S., Corey S.P., Stricker E.M., Kaplan B.B. (1992). Selective activation of nore-pinephrine-and epinephrine-secreting chromaffin cells in rat adrenal medulla. Am. J. Physiol. Regul. Integr. Comp. Physiol..

[19] Kastriti M.E., Kameneva P., Adameyko I. (2020). Stem cells, evolutionary aspects and pathology of the adrenal medulla: A new developmental paradigm. Mol. Cell. Endocrinol..

