# Supplementary material for: Chronic Ultraviolet Irradiation to the Skin Dysregulates Adrenal Medulla and Dopamine Metabolism In Vivo
Source: Antioxidants (Basel). 2021 Jun 7;10(6):920. doi: 10.3390/antiox10060920 (PMC8228565; doi:10.3390/antiox10060920)
Supplement: Supplementary file 1 [file antioxidants-10-00920-s001.zip › antioxidants-1209710-supplementary.pdf]

Supplementary Material

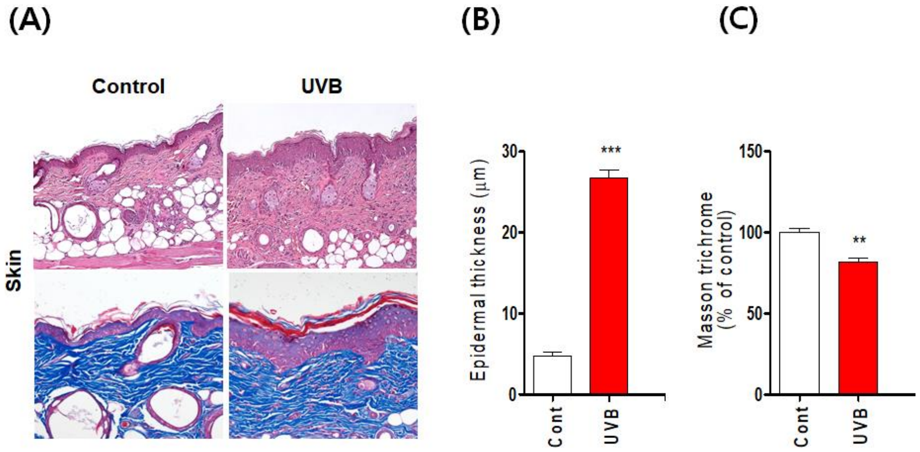

Supplementary Figure 1. Accelerated skin aging in mice induced by skin exposure to chronic excessive UVB irradiation. (**A**) Representative histological skin sections after chronic excessive exposure stained with hematoxylin & eosin (upper panels) and Masson's trichrome (lower panels). Changes in the epidermal thickness (**B**) and collagen fiber intensity (**C**) in the stained skin sections. Values are presented as the mean ± standard error of the mean. Statistical significance of differences is illustrated as follows: ** $p < 0.01$ and *** $p < 0.001$ compared with the control group.

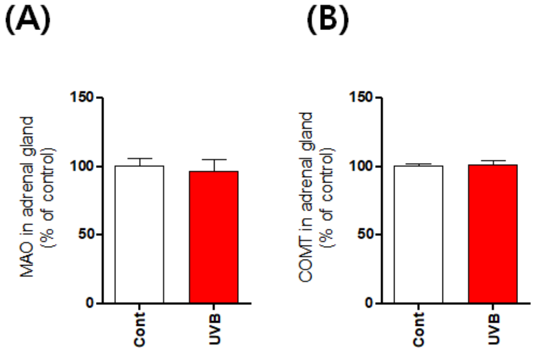

Supplementary Figure 2. Lack of changes in the levels of dopamine metabolism-related enzymes following mouse skin exposure to chronic excessive UVB irradiation. Monoamine oxidase (MAO) and catechol-O-methyltransferase (COMT) levels in the adrenal glands were quantified using ELISA kits. Values are presented as the mean ± standard error of the mean.

Supplementary Table 1. UVB irradiation schedule.

	**Mondays**	**Wednesdays**	**Fridays**
Week 1	100 mJ/cm^2	100 mJ/cm^2	120 mJ/cm^2
Week 2	120 mJ/cm^2	140 mJ/cm^2	140 mJ/cm^2
Week 3	160 mJ/cm^2	160 mJ/cm^2	180 mJ/cm^2
Week 4	180 mJ/cm^2	200 mJ/cm^2	200 mJ/cm^2
Weeks 5–10	220 mJ/cm^2	220 mJ/cm^2	220 mJ/cm^2
